# Anti-idiotypic antibody Ab2/3H6 mimicking gp41: a potential HIV-1 vaccine?

**DOI:** 10.1186/1753-6561-5-S8-P64

**Published:** 2011-11-22

**Authors:** Renate Kunert, Alexander Mader

**Affiliations:** 1 Department of Biotechnology, Institute for Applied Microbiology, BOKU – University of Natural Resources and Life Sciences, A-1190 Vienna, Austria

## Background and aims

Anti-idiotypic antibodies (Abs) represent an alternative vaccination approach in human therapy. This approach is based on the idiotype (Id) network theory postulated by Jerne describing the Ab (Ab1) – anti-idiotypic Ab (Ab2) – anti-anti-idiotypic Ab (Ab3) cascade stimulation. Specific anti-Id Abs serve as an “internal image” of the target antigen and can be used to induce Abs able to bind to the cognate antigen [1]. The anti-Id Ab Ab2/3H6 [2], developed at our Institute was generated in mouse and is directed against the human monoclonal Ab (mAb) 2F5, which broadly and potently neutralizes primary HIV-1 isolates [3]. Ab2/3H6 which has been characterized previously [4,5] is able to mimic the antigen recognition site of 2F5 and therefore it is suggested as a putative candidate for an HIV-1 vaccine.

We investigated the potential of Ab2/3H6 by immunization of Fab fragments and fusion proteins with interleukin 15 (IL15) and tetanus toxin (TT) tags as immune modulators. After three prime/boost administrations rabbit sera were purified and analyzed for 2F5-like specific Abs. Further, the 2F5-like Abs from the sera were enriched by affinity purification and characterized for their binding affinity to 2F5.

In an additional approach we applied different humanization methods to reduce the immunogenicity of the originally mouse derived Ab2/3H6. The mouse variable regions of Ab2/3H6 were subjected to three different humanization methods, namely resurfacing, CDR-grafting and superhumanization. Four differently humanized Ab2/3H6 variants were characterized for their binding affinity to 2F5 in comparison to the original Ab2/3H6.

## Results

**Figure 1:**
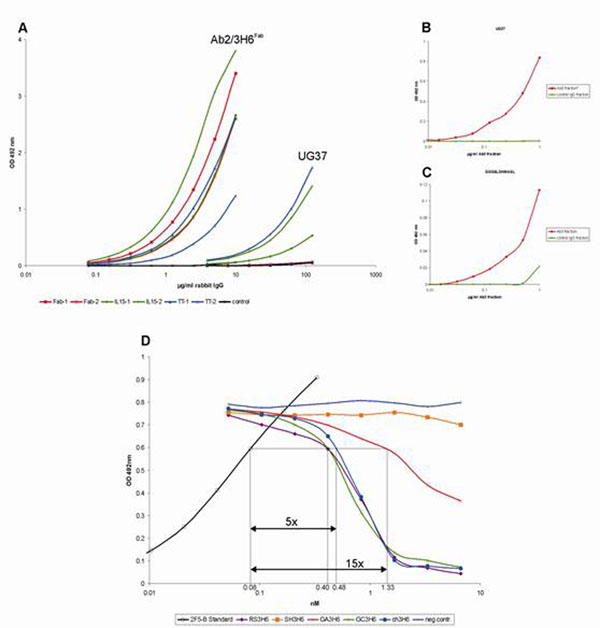
**(A)** Binding of purified rabbit IgGs immunized with different Fab preparations and control (pre-immun IgG fraction) to Ab2/3H6Fab and recombinant HIV-1 gp140 (UG37); **(B)** Binding of the Ab3 (2F5-like) Ab fraction purified from pooled rabbit IgG fractions to recombinant HIV-1 gp140 (UG37) and **(C)** synthetic 2F5 epitope (ELDKWA); **(D)** Competition ELISA. Biotinylated 2F5 (2F5-B) was allowed to complex with serial dilutions of Ab2/3H6 mutants and afterwards uncoupled 2F5-B was determined on an epitope pre-coated ELISA plate. The optical density resulting from binding of 2F5-B to the synthetic epitope GGGELDKWASL is plotted versus the logarithm of the concentration of Ab2/3H6 mutants.

To evaluate the humoral immune response of Ab2/3H6 we designed Ab2/3H6^Fab^ fusion proteins with IL15 and TT. Recombinant CHO cell lines were established and after protein purification New Zealand white rabbits were immunized with the Ab2/3H6^Fab^ variants. Ten days after the final boost sera were collected and analyzed for total rabbit IgG levels. After proteinA affinity purification of the sera the isolated rabbit IgGs were tested for Ab2/3H6^Fab^ and recombinant gp140 (UG37) specificity (Figure 1A). Further an affinity enrichment step using a UG37/ELDKWA column was performed and the obtained Ab3 fraction was tested on UG37 (Figure 1B) and additionally on the original 2F5 epitope ELDKWA (Figure 1C). Finally the Ab3 fraction was tested for binding affinity to the UG37 in a bio-layer interferometry assay which showed that the Ab3 fraction has a 6.6 fold reduced affinity towards UG37 compared to the mAb 2F5 (Table 1).

**Table 1:** Summary of k-values for binding to UG37

Samples [c=400nM]	kon [1/Ms]	koff [1/s]	KD [nM]
**mAb 2F5**	1.66 x10^04^	3.09 x10^-04^	18.65
**Ab3 fraction**	1.56 x10^04^	1.91 x10^-03^	122.90
**Control IgG fraction**	n.d.	n.d.	n.d.

For the humanization approach three different methods were chosen. The “resurfaced” variant (RS3H6) was developed by a computer model and surface exposed amino acids in the murine framework (FR) were substituted by residues usually found at equivalent positions in human Abs. The “superhumanized” form (SH3H6) was designed by structural homologies between the murine Ab2/3H6 CDRs and human germline CDRs. The most homologous human germline Ab was then used as acceptor FR. For the “CDR-grafted” variant two versions were expressed. An “aggressive” graft (GA3H6) harbouring less backmutations, making the grafted Ab more human-like and a “conservative” graft (GC3H6) with more backmutations. The different “reshaped” variable regions of Ab2/3H6 were expressed in CHO-cells as IgG1 molecules. The obtained “humanized” Ab variants were further characterized by competition ELISA (Figure 1D).

RS3H6 and GC3H6 showed a nearly identical slope of a concentration dependent 2F5 inhibition compared to the chimeric (ch) 3H6. RS3H6, GC3H6 or ch3H6 complexed 50 % of mAb 2F5 with a five fold molar excess. GA3H6 was able to bind 50 % of 2F5 in a 15 fold excess. In contrast, SH3H6 did not interact with 2F5 even in an 80 fold excess which is comparable to the negative control (unspecific IgG). The ELISA results were supported by binding affinity experiments with 2F5 in a bio-layer interferometry assay: RS3H6, GC3H6 and ch3H6 have similar binding affinity to 2F5 whereas GA3H6 has a 2-fold reduction in affinity.

## Discussion and conclusion

The Ab2/3H6 was generated to be used as an HIV-1 vaccine, based on the induction of 2F5-like Abs (Ab3s). First we did a “proof of concept” and immunized rabbits with different Ab2/3H6Fab fusion proteins to induce 2F5-like Abs. Immunochemical analyses showed that the use of IL15 and TT as immune modulators do not significantly enhance total rabbit IgG levels or the production of Ab2/3H6Fab specific Abs. However it could be demonstrated that UG37 binding Abs (Ab3s) were induced significantly (Figure 1A). Quantification of various purification steps revealed that only 0.7 % of the rabbit IgG fraction contained UG37/ELDKWA binding Abs. This affinity enriched Ab3 fraction shows significant binding to UG37 and reduced binding to the synthetic 2F5 epitope ELDKWA in ELISA (Figure 1B/C). Affinity binding studies showed that the obtained Ab3 fraction has only a 6.6-fold lower affinity to the recombinant UG37 protein compared to 2F5 (Table 1).

In a next step different humanization methods were evaluated. The “resurfaced” and the “conservative” grafted variants showed similar binding properties to 2F5 as the original Ab2/3H6 version, while the “aggressively” grafted Ab had a 2-fold lower affinity and the “superhumanized” form completely lost the ability to bind to 2F5.

We postulate that after humanizing the framework regions of Ab2/3H6 the immune response will be focused towards the paratope of the Ab2 and therefore enhance elicitation of Ab3 when administered during human therapy. Therefore affinity to 2F5 is not the crucial factor in evaluating the best candidate for a clinical study. Our results show that the Ab2/3H6 variants RS3H6 and GA3H6 would be suitable candidates for future studies.
